# ArcDrain: A GIS Add-In for Automated Determination of Surface Runoff in Urban Catchments

**DOI:** 10.3390/ijerph18168802

**Published:** 2021-08-20

**Authors:** Cristina Manchado, Alejandro Roldán-Valcarce, Daniel Jato-Espino, Ignacio Andrés-Doménech

**Affiliations:** 1 EGICAD Research Group, Universidad de Cantabria, 39005 Santander, Spain; cristina.manchado@unican.es; 2 GITECO Research Group, Universidad de Cantabria, 39005 Santander, Spain; alejandro.roldan@unican.es; 3 GREENIUS Research Group, Universidad Internacional de Valencia–VIU, Calle Pintor Sorolla 21, 46002 Valencia, Spain; 4 Instituto Universitario de Investigación de Ingeniería del Agua y Medio Ambiente (IIAMA), Universitat Politècnica de València, 46022 Valencia, Spain; igando@hma.upv.es

**Keywords:** automation, flood mitigation, geographic information system, land cover, peak flow, permeable surfaces, rational method, urban runoff

## Abstract

Surface runoff determination in urban areas is crucial to facilitate ex ante water planning, especially in the context of climate and land cover changes, which are increasing the frequency of floods, due to a combination of violent storms and increased imperviousness. To this end, the spatial identification of urban areas prone to runoff accumulation is essential, to guarantee effective water management in the future. Under these premises, this work sought to produce a tool for automated determination of urban surface runoff using a geographic information systems (GIS). This tool, which was designed as an ArcGIS add-in called ArcDrain, consists of the discretization of urban areas into subcatchments and the subsequent application of the rational method for runoff depth estimation. The formulation of this method directly depends on land cover type and soil permeability, thereby enabling the identification of areas with a low infiltration capacity. ArcDrain was tested using the city of Santander (northern Spain) as a case study. The results achieved demonstrated the accuracy of the tool for detecting high runoff rates and how the inclusion of mitigation measures in the form of sustainable drainage systems (SuDS) and green infrastructure (GI) can help reduce flood hazards in critical zones.

## 1. Introduction

Climate change and urban sprawl are increasing the frequency of natural hazards such as landslides, floods, and droughts [1,2]. The economic and human losses caused by floods have been especially severe during recent years. Following the trends observed during the last two decades, flood events in 2020 were 23% more recurrent and had 18% more fatalities, causing $51.3 billion losses worldwide [3]. Therefore, developing tools and methods to support the ex-ante identification of flood prone areas is becoming increasingly crucial for the urban planning of future societies.

The evaluation and eventual mitigation of these events is commonly approached with the support of geographic information systems (GIS), which have been highlighted as useful tools to help identify flood prone areas [4,5]. GIS enable handling a variety of spatial data, whose geoprocessing is essential to hierarchize a study site according to its sensitivity to urban flooding due to runoff accumulation. Moreover, the use of GIS to support surface runoff determination is a recurrent topic in the literature, especially when oriented to flood calculation.

In this sense, a vast array of research focuses on the overlaying of thematic layers to produce hazard maps [6,7,8,9,10,11,12,13]. These studies resort to multi-criteria decision analysis (MCDA), especially in the form of weighted sum techniques, to aggregate a series of factors expected to contribute to flooding, such as the slope, land cover, or precipitation. Many other investigations couple GIS with different stormwater computer models, such as SWMM (US EPA, Washington, DC, USA) [14,15], HEC-HMS (U.S. Army Corps of Engineers, Washington, DC, USA) [16,17], SWAT (USDA Agricultural Research Service (USDA-ARS) and Texas A&M AgriLife Research, Maryland and Texas, USA) [18,19], or MIKE FLOOD (DHI Group, Inc., New York, NY, USA) [20,21]. These approaches use GIS to produce a variety of types of spatial information that then functions as inputs in the stormwater models, which in turn serve to simulate rainfall–runoff processes.

Although the development of standalone GIS tools for surface runoff determination is not very common, some precedents can be found in the recent literature. This is the case of Itzï [22], a 2D numerical model integrated in GRASS via Python. The methods used by Itzï for computing rainfall–runoff are simplified versions of the shallow water equations, the damped partial inertia equation, and the Green–Ampt model. Python is the most recurrent programming environment to automate the integration of hydrological tasks in GIS, as demonstrated by IDW-Plus [23], an ArcGIS toolbox that enables estimating runoff potential using distance-weighted metrics. RUFIDAM [24] is another tool combining Python with ArcGIS (Esri, West Redlands, CA, USA), whose integration results in a 1D model capable of simulating urban flood inundation based on water balance and topography. Despite these tools being efficient for calculating runoff, their infiltration modules (if any) only focus on the underlying soil, without accounting for specific parameters related to surface imperviousness. Consequently, their potential to estimate the effects of future adaptation measures is limited.

Instead of focusing on the spatial implementation of theoretical hydrological methods, other approaches concern the development of graphical user interfaces (GUIs) to run existing stormwater computer programs in GIS environments. For instance, WetSpa-Urban [25] was designed as an event-based and continuous rainfall–runoff model that links GIS and SWMM via Python. In a similar vein, DrainGIS [25] emerged as a plug-in built using Python to incorporate the infiltration and routing methods available in SWMM into QGIS. These tools, which are variants of the proprietary software PCSWMM [26], entail the same degree of parametrization and complexity as the original stormwater model. As such, they are not especially suitable for rapid and computationally efficient runoff determination.

Finally, unlike the trend observed in the above referenced models, whereby they contain different modules to account for the main hydrological processes involved in the transformation of rainfall into runoff, there is a group of simpler tools that only use the soil conservation service curve number (CN) method to compute runoff from rainfall data [27,28,29,30]. These applications are exactly the opposite of the former, since they are distinguished by their accessibility and straightforwardness. However, their effectiveness for accurate runoff estimation is narrowed by the lack of methods for calculating flow routing. Consequently, they are inadequate to compute flow accumulation and, by extension, assess flood susceptibility.

Given the characteristics of the GIS-based tools for runoff determination found in the recent related literature, this research sought to develop ArcDrain, an ArcGIS add-in for automating the rational method. ArcDrain was intended to act as a bridge between the weaknesses and strengths observed in previous tools, such that it provides a simple and yet accurate means to compute urban peak runoff. This add-in was built with ArcGIS Pro SDK for Microsoft .NET and coded with C#, a compiled language. Using the native code of the target machine results in a faster and more efficient performance, which contrasts with the course of action widely adopted in the literature, whereby Python, an interpretable language, is preferred. Moreover, the direct dependence of the rational method on the land cover configuration of catchment areas facilitates assessing the impact of imperviousness on runoff and how the implementation of permeable surfaces can contribute to reducing it. These aspects were tested using the city of Santander (northern Spain) as a case study.

## 2. Materials and Methods

**Figure 1:**
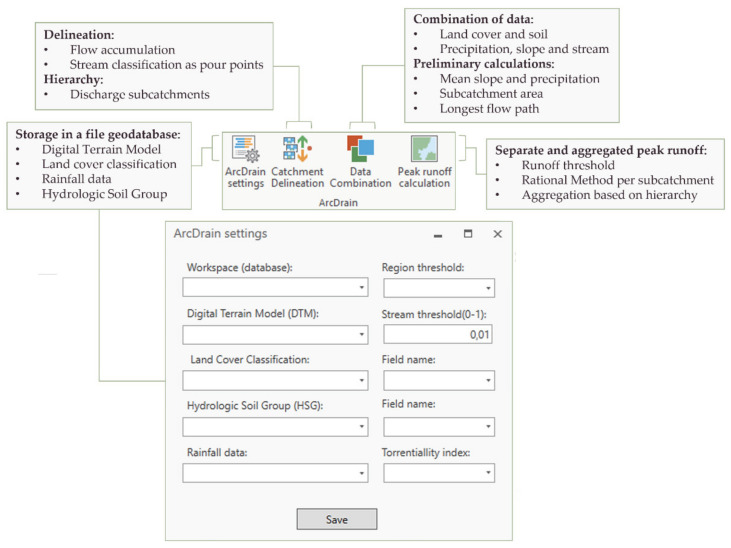
Breakdown of the ArcDrain ribbon into four modules, and their main tasks.

ArcDrain was developed as an add-in for ArcGIS Pro (Esri, West Redlands, CA, USA) [31]. It was structured in four modules as depicted in Figure 1. The first of them concerned the setup of the add-in and the storage of layers in a file geodatabase. Then, a series of operations were carried out to realize the delineation and hierarchy of the study catchment based on the path taken by water after a rainfall event. Third was undertaking some pre-processing tasks, whereby different layers were combined to be used in the last module, which also required a series of preliminary calculations to yield mean values per subcatchment. Finally, the rational method was applied to determine peak runoff in each subcatchment in the study area (Santander, Spain), thereby enabling their subsequent aggregation, to obtain accumulated runoff rates. The specifics of each module are provided below in detail.

### 2.1. ArcDrain Settings

The data required to use the ArcDrain add-in includes four inputs, as summarized in Table 1. Due to the urban context of the case study, the sewer network was also considered as an input at the beginning of the investigation. However, an inspection of the geometry and size of the pipes and manholes in the layer available at the Open Data repository of the case study revealed multiple errors, missing values, and disconnections questioning its validity. Nevertheless, even if omitting the sewer network, ArcDrain still enables the prioritized location of flood-prone zones through the calculation of peak runoff in the subcatchments included in the study area. Although the extent and depth of flooding varies, depending on whether sewer networks are considered or not, the hierarchy of flood maps is expected to be similar in both scenarios [32]. As such, ArcDrain can serve to provide a numerical comparison of the flood susceptibility of urban subcatchments, based on their values of accumulated peak runoff.

Another factor hindering the consideration of the sewer network as an input was its limited availability, since detailed information in this respect is often privately stored by the entities in charge of urban water management. Instead, the datasets shown in Table 1 are accessible worldwide via different sources, which guarantees the replicability of the proposed approach. At a European level, a digital elevation model (DEM) with a 25 m resolution is available from the Copernicus Land Monitoring Service [33]. The same source provides pan-European land cover and land use data for urban areas, through the Urban Atlas [34]. Soil type can be obtained from the hydrogeological map prepared by the Federal Institute for Geosciences and Natural Resources [35], whilst daily precipitation records are compiled in the European Climate Assessment and Dataset [36].

These data must be processed to different extents prior to their inclusion in ArcDrain. In the case of the DEM, this simply consists of clipping to the boundaries of the study area. The land cover map requires a field including an equivalent classification to the 3-digit code scheme presented by the Urban Atlas or the Corine Land Cover, since they fit the breakdown and level of detail required to apply the rational method. In a similar vein, soil types must be classified according to the hydrologic soil groups (HSGs) [24], whose runoff potential ranges from low (A) to high (D), when thoroughly wet, and including moderately low (B) and moderately high (C) intermediate levels. These infiltration capacities refer to the following textures: sandy and silty sand (A); sandy loam, loam, sandy clay loam, and silty loam (B); clay loam, silty clay loam, and sandy clay (C); and clay (D), respectively.

Precipitation is the input requiring more thorough preparation, since it should be acquired in the form of daily records, to enable producing extreme storms associated with values of annual maximum daily rainfall for different return periods. Since ArcDrain was conceived to be used at the scale of entire cities, these values must be spatially interpolated to result in a continuous precipitation raster layer, in order to provide a more accurate representation of the variability of this parameter. Once prepared, all the layers associated with the data shown in Table 1 were stored in a file geodatabase linked to the add-in to facilitate its use in subsequent modules.

### 2.2. Catchment Delineation

The first step in this module consisted of delineating the study area into a series of subcatchments based on surface water flow paths. This was carried out by automating the application of a series of ArcGIS tools from a digital elevation model (DEM). To this end, the first task was removing imperfections from the DEM using the ‘Fill’ tool, performing as a precursor for the determination of a flow direction map [37]. Next was computing the number of cells upstream of each cell in the study grid through the ‘Flow Accumulation’ tool [38]. The layer stemming from this task and the flow direction map were used to create a stream network formed by cells with at least 1% of upstream cells flowing to them. This resulted in a grid with unique values for each stream segment, thereby enabling its use as pour points and subsequent combination with the flow direction map to delineate the study catchment using the ‘Watershed’ tool.

Each pixel in the resulting raster grid was unequivocally identified with its associated subcatchment, storing all the data in a numeric internal collection. In parallel, the initial and final nodes of each segment in the stream network were identified using the Shreve stream ordering method in the ‘Stream Order’ tool [39], which also indicated the subcatchment they belonged to. This information enabled creating a search algorithm to identify the discharge point associated with each subcatchment, leading to a hierarchy in the study area based on the number of tributaries of each stream.

### 2.3. Data Combination

**Figure 2:**
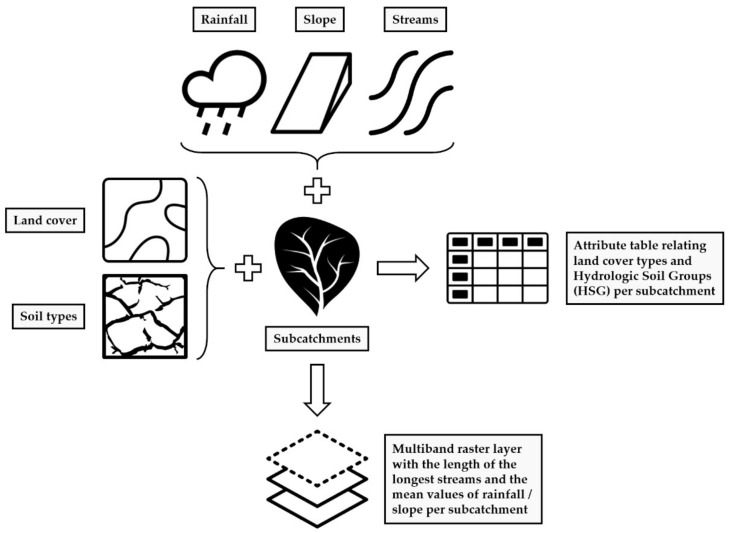
Pre-processing tasks involved in the data combination module of ArcDrain. Icons created by Daan, Douglas Machado, DPIcons, Sumana Chamrunworakiat, Brown_Iconic, Andreas, iconixar, and Andrejs Kirma from the Noun Project.

This module included a series of pre-processing tasks to handle some of the variables involved in the determination of peak runoff, as illustrated in Figure 2. On the one hand, discrete data such as land cover and soil types were combined with the catchment layer to result in an attribute table containing the relationships among these variables. On the other hand, a multiband raster layer was produced by combining continuous data such as the precipitation, slope, and stream network with the subcatchments. This procedure enabled preparing all the data required to apply the rational method in the last module of the ArcDrain add-in.

Furthermore, a set of additional tasks was run to prepare some data to be used in the last module of ArcDrain. Hence, the mean rainfall across the catchment was determined by iterating the values of annual maximum daily precipitation across each subcatchment. In a similar vein, the outputs derived from the ‘Flow Length’ tool were used to compute the length of the longest stream in each subcatchment. Finally, the ‘Slope’ geoprocessing tool was applied to calculate the steepness at each cell of the DEM. This raster layer was then used to store the mean slope in the subcatchments delineated in the previous module.

### 2.4. Peak Runoff Calculation

Since this study was framed within a Spanish research project, runoff in the study area was determined as specified in the 5.2-IC Surface Drainage standard [40] established by the Spanish Ministry of Transport, Mobility, and Urban Agenda. This standard suggests using the rational method [24], as shown in Equation (1), to calculate peak runoff in catchments with an area less than 50 km^2^.

(1) $$Q_{T}=\frac{I\cdot C\cdot A\cdot K_{t}}{3.6}$$

where $Q_{T}$ (m^3^/s) is the peak runoff corresponding to a return period T at the discharge point of the catchment, I (mm/h) is the rainfall intensity associated with a return period T and a storm event duration equal to the time of concentration $t_{c}$ of the catchment, C (dimensionless) is the mean runoff coefficient in the catchment, A (km^2^) is the area of the catchment, and $K_{t}$ (dimensionless) is the uniformity coefficient in the temporal distribution of precipitation. Despite its simplicity, this method has been argued to provide an effective compromise between theory and data availability [41], which is in line with the premise of ArcDrain and the accessibility of the inputs listed in Table 1. In addition, many standards and design manuals for urban drainage consider the rational method for the estimation of peak flows [42,43,44].

Aggregated runoff rates in the study area were computed based on the dependencies among subcatchments, whose individual values of peak runoff were determined through the application of Equation (1). Except for A, which was measured from the ratio of the number of pixels in each subcatchment to the total pixel count and the resolution of the raster layer, the remaining terms in Equation (1) required more elaborate calculations, as described below.

#### 2.4.1. Rainfall Intensity

This parameter represents the average rainfall rate associated with a duration equal to the time of concentration for a return period. Once a return period is set and the time of concentration is calculated, rainfall intensity (I) should be determined from intensity–duration–frequency (IDF) curves of a nearby weather station if local data is available [45].

In the absence of these data, below is presented an approach to compute this variable as the product of $I_{d}$ (mm/h) by $F_{int}$ (dimensionless), which represent average daily corrected rainfall intensity and the intensity factor, respectively. The former was obtained using Equation (2).

(2) $$I_{d}=\frac{P_{d}\cdot K_{A}}{24}$$

where $P_{d}$ stands for the maximum annual daily precipitation associated with a certain return period and $K_{A}$ is a reducing factor of precipitation, according to catchment area (A). It takes a value of 1 in those cases in which A<1 km^2^, otherwise being determined as indicated in Equation (3).

(3) $$K_{A}=1-\frac{\log_{10}A}{15}$$

The second term involved in the calculation of I, denoted as intensity factor ($F_{int}$), accounts for the torrentiality of precipitation in the study area. Equation (4) provides a generic expression for determining the intensity factor from the torrentiality index ($I_{1}/I_{d}$).

(4) $$F_{int}={\left(\frac{I_{1}}{I_{d}}\right)}^{3.5287-2.5287\cdot t_{c}^{0.1}}$$

where $I_{1}$ represents the hourly rainfall intensity. The whole term ($I_{1}/I_{d}$) represents the torrentiality index of precipitation and depends on the geographic area in which the study takes place. In the case of Spain, it ranges from 8 in the northwest and southern regions to 11 across the Mediterranean Sea. $t_{c}$ is the time of concentration, which refers to the minimum time required from the onset of a rainfall event for the entire catchment to be contributing to runoff generation at its discharge point [24]. Its calculation was carried out using Equation (5).

(5) $$t_{c}=0.3\cdot L_{s}^{0.76}\cdot J_{s}^{-0.19}$$

where $L_{s}$ is the length of the longest stream in the catchment, and $J_{s}$ is the mean slope of such a stream. Although this formula was derived from time series data collected in several natural basins, the incorporation of the runoff coefficient in Equation (1), as described below, enabled accounting for the degree of urbanization of the catchment. Furthermore, a recent analysis of the most widely used time of concentration formulas in the literature [46] revealed that Equation (5) was in the group showing less uncertainty. This uncertainty was mainly attributed to the stream threshold. In this case, this parameter was set at 1%, since lower values resulted in very small subcatchments, only formed by a few pixels. Therefore, according to the study carried out by Azizian and Shokoohi (2014) [47], this was the most restrictive possible threshold in terms of resulting in higher peak runoff rates.

#### 2.4.2. Runoff Coefficient

The runoff coefficient (C) is a factor relating the amount of runoff produced in a subcatchment to the amount of precipitation received. Common values for C are usually tabulated according to the slope, land use/cover, and/or soil permeability [48]. However, this approach assumes that rainfall occurs simultaneously across the entire catchment area by disregarding $K_{A}$. The consideration of this parameter led to obtaining a more realistic characterization of C, which was determined through the application of Equation (6).

(6) $$\begin{array}{c} C=\frac{\left(\frac{P_{d}\cdot K_{A}}{P_{0}}-1\right)\cdot \left(\frac{P_{d}\cdot K_{A}}{P_{0}}+23\right)}{{\left(\frac{P_{d}\cdot K_{A}}{P_{0}}+11\right)}^{2}},\;\;\;\;if\;P_{d}\cdot K_{A}>P_{0}\\ \;\;\;\;\;\;C=0,\;\;\;\;if\;P_{d}\cdot K_{A}\leq P_{0}\; \end{array}$$

where $P_{0}$ is the runoff threshold, which is the minimum amount of precipitation required for runoff generation. Therefore, it depends on a series of parameters that affect runoff, including the permeability of the land coverage, its mean slope, the infiltration capacity of the underlying soil, and the cropping method used, when appropriate. As such, the values of $P_{0}$ are tabulated based on these four parameters, as shown in Table 2.

#### 2.4.3. Uniformity Coefficient in the Temporal Distribution of Precipitation

The most basic and common expression used for the rational method omits this coefficient from Equation (1). This means that the temporal distribution of precipitation is often assumed to be uniform. For the sake of comprehensiveness, this parameter was taken into account and computed as formulated in Equation (7), based on the time of concentration ($t_{c}$) of the catchment.

(7) $$K_{t}=1+\frac{t_{c}^{1.25}}{t_{c}^{1.25}+14}$$

$K_{t}$ is the last parameter required to apply the rational method as formulated in Equation (1). Using the four datasets listed in Table 1 as its only input requirements, the ArcDrain add-in enables automating all the calculations behind the four modules summarized in Figure 1, including the determination of every term and coefficient defined from Equation (2) to Equation (7).

## 3. Results and Discussion

ArcDrain was tested using the city of Santander, Cantabria (northern Spain), as a case study. The Köppen–Geiger classification of Santander is Cfb, corresponding to an oceanic climate. This type of climate means constant moisture and narrow ranges of temperature throughout the year, including cool winters and temperate summers. These conditions result in mean values of annual precipitation of 1200 mm, which supports the interest in analyzing this city. Furthermore, the city council released a Municipal Emergency Plan to account for natural, anthropogenic, and technological risks [49], among which flood risk was one of the main threats identified due to the existence of several areas across the city proving to be especially sensitive to runoff accumulation.

**Figure 3:**
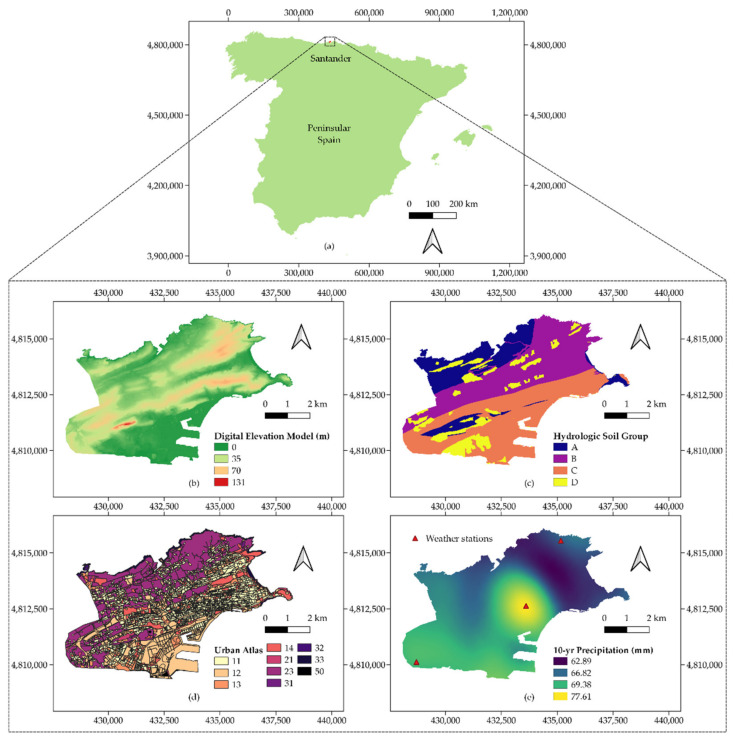
Location of the case study and inputs, (**a**) situation map of the city of Santander, (**b**) digital elevation model (DEM), (**c**) hydrologic soil group (HSG), (**d**) urban atlas land cover map, and (**e**) maximum annual daily precipitation for a return period of 10 years.

Figure 3 represents the location of Santander and the four inputs required by the menu emerging after clicking on the ArcDrain’s “Setup: data preparation” button: digital elevation model (DEM), hydrologic soil group (HSG), land cover, and extreme precipitation. The DEM was acquired from the Spanish Geographic Institute with a 5 m resolution, obtained by interpolation from LiDAR flights of the National Plan for Aerial Orthophotography (PNOA) [50]. Its reference system is ETRS89, projected in the Universal Transverse Mercator (UTM) zone 30N, with orthometric heights. The HSG was calculated according to the permeability of the soil types included in the 1:25,000 geological map available at the mapping service of the region [51], whilst land cover was provided by the Urban Atlas at a 1:10,000 scale [34].

Precipitation was the input requiring more pre-processing work, since it stemmed from a 1 km^2^ grid of points, with values of daily rainfall across Cantabria [52], the region of which Santander is the capital. This grid was obtained from 148 weather stations distributed throughout the area, with records between 1950 and 2003. The resulting data were fitted to a list of probability functions to obtain values of annual maximum precipitation, which were then spatially interpolated using ordinary kriging to yield the map depicted in Figure 3e [53]. The unevenly distributed and limited number of stations available in Santander explains the shape of the precipitation map. Furthermore, the peak found in the city center is in line with the findings of Baik et al. (2001) and Han et al. (2014) [54,55], who argued that built-up areas can boost precipitation through increased moist convection.

**Figure 4:**
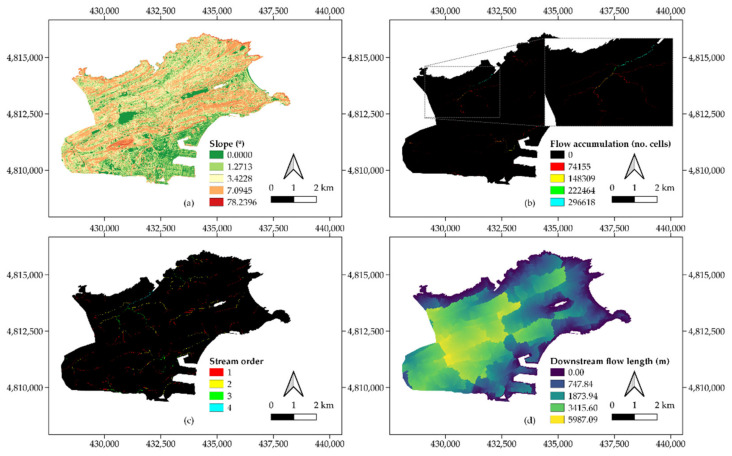
Intermediate output layers, (**a**) slope, (**b**) flow accumulation, (**c**) stream order, and (**d**) downstream flow length.

The DEM was the base layer required to generate a variety of intermediate outputs preceding the calculation of the catchment area using the “Catchment delineation” button. The process of filling the depressions in the DEM revealed the role played by some water bodies, such as the artificial lake of the Las Llamas Park, whose presence was found to lead to some inconsistencies in terms of flow direction. As such, the original DEM layer was masked according to the location of these water bodies to avoid these errors. The addition of these gaps in relation to Figure 3b can be observed in some southern and central-eastern regions in the slope map represented in Figure 4a. The aspect of the flow accumulation map indicated the sensitivity of the zoomed area in Figure 4b, which was especially susceptible to receiving large runoff volumes due to the orography in upstream cells.

The definition of the stream network associated with the flow accumulation map and its subsequent ordering yielded the layer shown in Figure 4c. This map provided insight into the hierarchy of the flow circulation paths across the study area. Depending on their slope and land cover condition, high order areas might eventually turn into flood prone zones. Stream order was also related to the output in Figure 4d, which concerns the flow length in each cell across the city. In accordance with the DEM and the flow accumulation map, those zones resulting in long downstream flow paths were associated with high values of elevation (Figure 3b).

The classification of streams as pour points depicted in Figure 4c enabled both the delineation of Santander into 247 subcatchments and the determination of their hierarchy according to precedence relationships. Once all the catchment-related calculations were carried out, the “Data combination” step was run as the next task in the execution of the ArcDrain add-in. This included the computing of mean values of slope and precipitation per subcatchment, whose mean area amounted to 0.137 km^2^. In addition, the longest flow path in each subcatchment was calculated from the values of flow length in Figure 4d.

**Figure 5:**
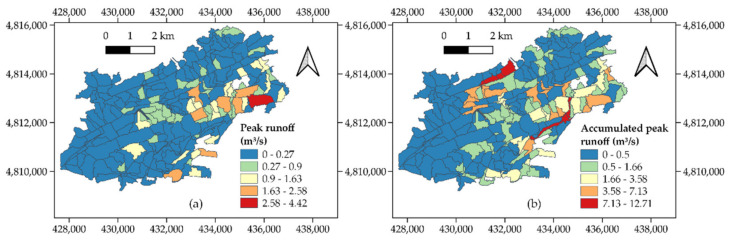
Values of (**a**) individual peak runoff (m^3^/s) and (**b**) accumulated peak runoff (m^3^/s) in the subcatchments.

Then, the “Peak runoff calculation” module was run to apply the rational method in the subcatchments, as formulated in Equation (1), and the remaining equations were derived from it. This resulted in the map represented in Figure 5a, which suggested a concentration of subcatchments with high values of peak runoff in the city centre. These subcatchments were associated with the greatest presence of built-up surfaces in the city (Figure 3d), whose impermeability might favour high runoff rates. The consideration of dependencies among subcatchments led to the values of accumulated peak runoff shown in Figure 5b. Although the city centre and its surroundings remained one of the most critical areas, this map emphasized the impact of the flow accumulation and stream order maps (Figure 4), whereby flood prone zones emerged in the northwest of the city due to the addition of flow routing processes among subcatchments to the calculations.

**Figure 6:**
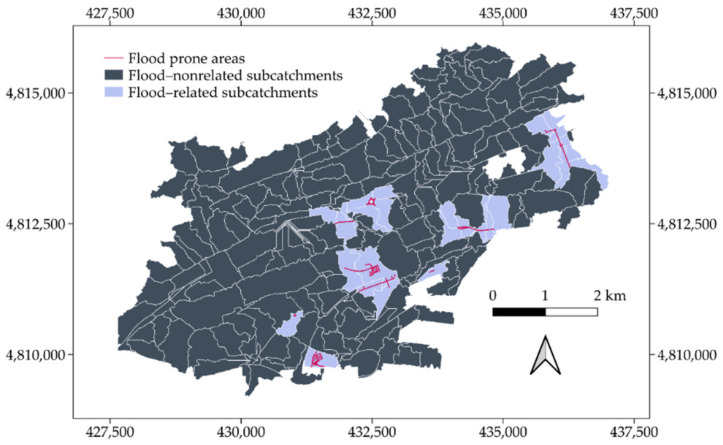
Overlap between the subcatchments delineated using ArcDrain and the flood prone areas highlighted by the City Council of Santander.

The validation of the results achieved in Figure 5 started by overlapping the flood risk areas identified by the city council in its Municipal Emergency Plan with the layer containing the geometrical arrangement of the subcatchments. Then, both inputs were intersected to identify which subcatchments were spatially coincident with these areas. Hence, those subcatchments intersecting any flood prone area were separated from the others, as illustrated in Figure 6. A visual inspection of Figure 5b and Figure 6 reveals a lack of agreement with regards to the group of subcatchments located in the north of the city. The latter achieved high accumulated runoff rates according to ArcDrain; however, this area was not considered a priority by the city council in terms of flood risk. This is due to its demographic characteristics, which include a low population density, as well as the limited existence of services and facilities that would be affected by floods. Thus, the interest in mitigating these events in such an area is reduced in comparison with more vulnerable zones.

**Figure 7:**
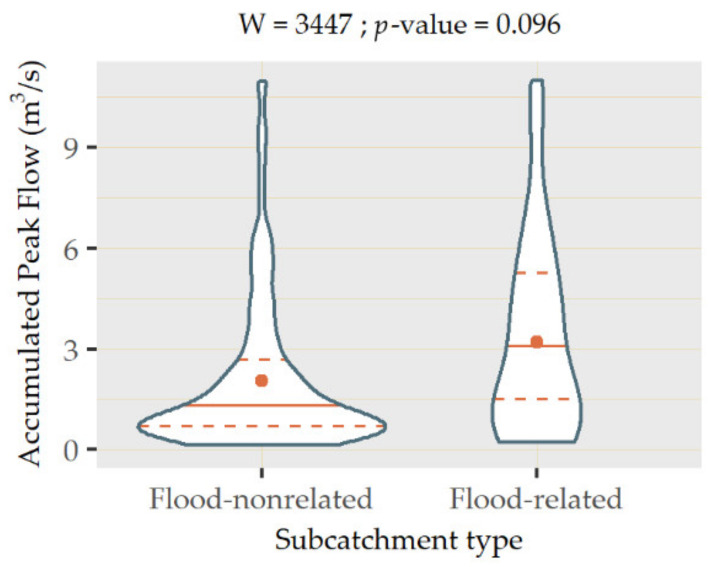
Violin plot of the values of accumulated peak flow in flood-related and flood-nonrelated subcatchments.

Nevertheless, although some of the subcatchments not intersecting flood prone areas achieved high accumulated peak flow rates, as shown in Figure 7, the mean and quantiles of the flood-related subcatchments were higher. To validate whether the values of accumulated peak flow in both types of subcatchments significantly differed from each other, statistical tests were applied. The type of test used was determined according to the distribution of the datasets associated with both groups of subcatchments. The results of the Shapiro–Wilk test indicated that their *p*-values were almost 0 in both cases, which suggested that none of the groups followed a normal distribution.

As such, a Mann–Whitney–Wilcoxon test (W) was applied to compare both types of subcatchments. The *p*-value yielded by the test was 0.096, demonstrating the existence of significant differences in accumulated peak flow in both groups for a significance level of 0.100. This output provided statistical evidence of the higher flood susceptibility of the subcatchments intersecting the areas identified by the City Council of Santander, which enabled verifying the reliability of ArcDrain. This validation approach is in line with the philosophy of the add-in, since one of its main premises was to be undemanding in terms of input requirements. In contrast with field measurements, whose availability can be rather limited, categorical or binary data on the existence of flooding can be acquired more easily or even produced ad hoc by carrying out surveys to get insight into past frequency trends in the study area.

**Figure 8:**
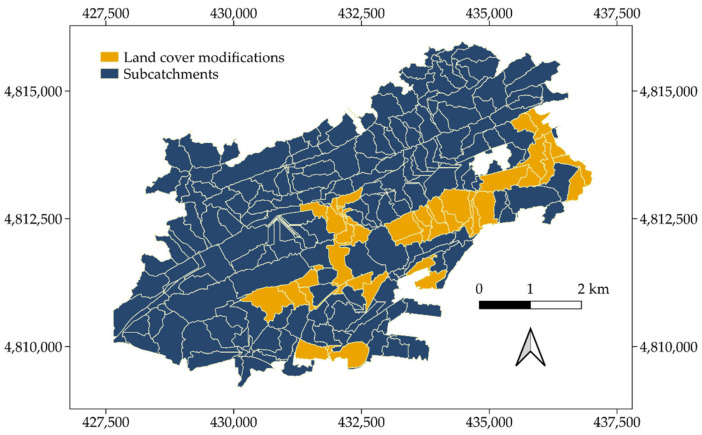
Shortlisted subcatchments to test different land cover modifications for reducing runoff accumulation.

Once validated under baseline conditions, the usefulness of ArcDrain was further explored by designing a series of scenarios aimed at reducing runoff accumulation. This was accomplished by replacing the following built-up land cover classes with permeable surfaces: discontinuous urban fabric (code 112) with green infrastructure (GI) (code 141); both continuous urban fabric (code 111) and industrial, commercial, public, military, and private units (code 121) with discontinuous very low-density urban fabric (code 112); and roads (code 122) by permeable pavements. Based on previous studies [56,57], the latter was assumed to have a runoff threshold equivalent to that of discontinuous very low-density urban fabric. These substitutions were applied in different proportions to those subcatchments having especially high values of individual peak flow and being connected to the flood prone areas depicted in Figure 6. Thus, four scenarios emerged in which 10%, 25%, 50%, and 100% of the surfaces associated with the abovementioned land covers were replaced in the subcatchments highlighted in Figure 8.

**Figure 9:**
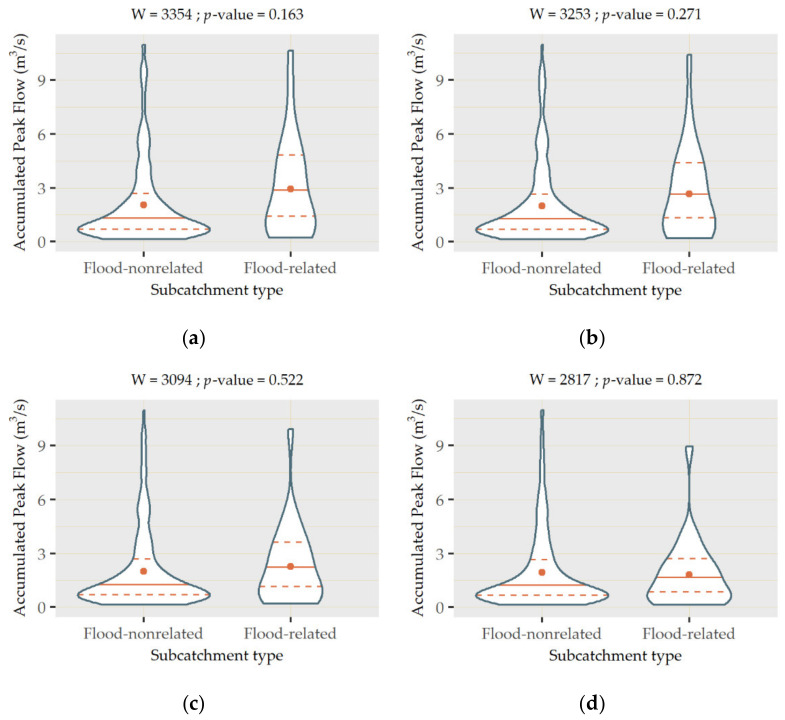
Violin plots of the values of accumulated peak flow in flood-related and flood-nonrelated subcatchments when substituting different proportions of built-up land cover classes with permeable pavements and green infrastructure: (**a**) 10% replacement, (**b**) 25% replacement, (**c**) 50% replacement, and (**d**) 100% replacement.

As a proof of the accuracy of this shortlisting process, a comparison between Figure 5a and Figure 8 reveals a strong agreement between subcatchments reaching high peak runoff rates and those selected for replacement purposes. ArcDrain was re-run with the changes included in the new scenarios. The *p*-values derived from the application of the Mann–Whitney–Wilcoxon test were 0.163, 0.271, 0.522, and 0.872 for the 10%, 25%, 50%, and 100% changes, respectively, as shown in Figure 9. All these values were considerably above any significance level commonly used in the literature, which demonstrated the effectiveness of the proposed measures. In accordance with these outputs, the violin plots produced for these new scenarios (Figure 9) reflect an increased concentration of values at their bottom, as the percentage of built-up surfaces replaced was higher.

This trend was supported by the median value reached by the new scenarios, which was progressively reduced from 2.447 to 2.296, 2.180, 2.038, and 1.663 m^3^/s in the flood-related subcatchments. Instead, the median of the other subcatchments remained generally unaltered, to the extent that it was constant (1.254) for the initial, 10% and 25% scenarios, and only varied when 50% (1.185) and 100% (1.025) of the built-up classes were replaced. This fact proved how accurate the strategic selection of subcatchments for replacement (Figure 8) was, since their effects were almost limited to the targeted flood prone areas.

To further demonstrate the benefits of increased permeability for runoff reduction, an outlier analysis of the values of accumulated peak runoff in the flood-prone areas was conducted for all the scenarios considered. In particular, Dixon’s test was chosen due to its usefulness for small sample sizes [58]. The *p*-values returned by the test were 0.351 and 0.152 for the initial and 10% replacement scenarios, which suggests that there were so many subcatchments with high values of accumulated peak flow that the existence of outliers was prevented. Instead, the *p*-values for the remaining scenarios were 0.036 (25%) and 0.000 (50% and 100%), indicating the presence of an outlier in each case. This means that most of the subcatchments identified as flood prone areas by the City Council of Santander had reduced values of accumulated peak flow in comparison with these outliers.

In view of all these results, a strategy to improve flood resilience in Santander might consist of replacing 25% of the roads and densely urbanized areas of the subcatchments highlighted in Figure 8 by permeable pavements and GI. These actions would contribute to attenuating peak runoff rates by enhancing the percolation capacity of the surfaces. Furthermore, the proposed measures could provide a variety of additional benefits aimed at urban regeneration, such as thermal regulation, improved aesthetics, air pollution control, or biodiversity values. As such, their adoption could spearhead future urban plans seeking to ensure the sustainability of cities in the context of climate and land cover changes.

## 4. Conclusions

This research designed, tested, and validated a GIS add-in called ArcDrain, which was aimed at facilitating the calculation of peak flow rates in urban catchments. ArcDrain is methodologically based on the rational method, whose simplicity and ease of application are completely in line with the idea of creating a tool to enable the rapid determination of surface runoff. The inputs required for running it only include a digital elevation model (DEM), a geologic or lithostratigraphic map, a land cover map, and a layer with the values of precipitation in the study area. These data can be obtained from continental or global open access repositories, which guarantees the applicability of ArcDrain at a worldwide scale.

The results achieved using Santander (northern Spain) as a case study highlighted the accuracy of the add-in, by fitting the flood-prone areas identified by its city council. A statistical analysis revealed the existence of significant differences between the values of accumulated peak runoff in the subcatchments adjacent to these vulnerable areas and the others. Unlike continuous GIS-based models for flood prediction, the proposed approach established interdependencies among the subcatchments forming the study area, which is very useful for identifying those locations that contribute the most to runoff accumulation. In turn, this facilitates the implementation of mitigation measures at strategic sites, to boost their effectiveness.

In this vein, part of the roads and built-up surfaces found in subcatchments flowing at high rates to the flood-prone areas identified by the city council were substituted by permeable pavements and green infrastructure, respectively. These modifications helped reduce the runoff coefficient of those subcatchments, thereby contributing to attenuating the accumulation of runoff. The testing of several replacement ratios revealed that 25% was the threshold from which the differences between flood-related and non-related subcatchments ceased to be statistically significant.

This fact emphasized the effectiveness of sustainable drainage systems (SuDS) and green infrastructure (GI) in attenuating the sealing of urban surfaces, which provides a solution to deal with the increasingly high threats posed by urbanization and climate change. As such, urban planning should be oriented towards promoting the implementation of these kinds of practices, emphasizing their connectiveness to make the most of their benefits and trying to replicate the hydrological cycle as much as possible. ArcDrain emerges as a tool to boost this approach by facilitating urban water management through the identification of strategic locations to implement flood mitigation measures.

The main limitations of this study relate to its constrained geographical extent, the lack of flow field measurements for calibration purposes, and the reduced customizability of the add-in concerning data entry. Consequently, follow-up research to further prove the usefulness of ArcDrain should consider its application to other regions with different climate characteristics. In addition, its accuracy should also be tested using quantitative measurements of surface water flow, in order to ensure its precision when contrasted with all types of validation data. Finally, the design of the add-in can also be improved to be more flexible and adaptive to the specifics of case studies, in terms of data availability.

## Figures and Tables

**Table 1:** Summary of datasets required to run the ArcDrain add-in.

Data	Type	Preparation
Digital elevation model	Grid	Clip to the limits of the study area
Land cover map	Polygon	Classification according to a 3-digit code
Hydrogeological map	Polygon	Classification based on runoff potential
Daily precipitation	Point	Calculation of extremes and interpolation

**Table 2:** Excerpt of the values adopted to determine the runoff threshold ($P_{0}$). Adapted from [40].

Code	Land Cover	Cropping Method	Slope (%)	Hydrologic Soil Group (HSG)			
				A	B	C	D
111	Continuous urban fabric	-	-	1	1	1	1
141	Green urban areas	-	-	53	23	14	10
212	Irrigated herbaceous crops	S ^1^	≥3	37	20	12	9
212	Irrigated herbaceous crops	C ^2^	≥3	42	23	14	11
212	Irrigated herbaceous crops	S/C	<3	47	25	16	13
311	Broad-leaved forest	-	-	90	47	31	23
331	Beaches and dunes	-	-	152	152	152	152
412	Peat bogs and meadows	-	-	248	99	25	16
522	Estuaries	-	-	0	0	0	0

^1^ S: Strip cropping; ^2^ C: Contour farming.

## Data Availability

Publicly available data were used in this study. The specific sources from which they stem are mentioned throughout the article.
